# Upregulation of Matrix Metalloproteinase-9 Protects against Sepsis-Induced Acute Lung Injury via Promoting the Release of Soluble Receptor for Advanced Glycation End Products

**DOI:** 10.1155/2021/8889313

**Published:** 2021-02-10

**Authors:** Hui Zhang, Yan-Fei Mao, Ying Zhao, Dun-Feng Xu, Yan Wang, Chu-Fan Xu, Wen-Wen Dong, Xiao-Yan Zhu, Ning Ding, Lai Jiang, Yu-Jian Liu

**Affiliations:** ^1^Department of Anesthesiology and Surgical Intensive Care Unit, Xinhua Hospital, Shanghai Jiaotong University School of Medicine, Shanghai 200092, China; ^2^School of Kinesiology, The Key Laboratory of Exercise and Health Sciences of Ministry of Education, Shanghai University of Sport, Shanghai 200438, China; ^3^Department of Physiology, Navy Medical University, Shanghai 200433, China; ^4^Department of Anesthesiology, Shandong Provincial Third Hospital, Cheeloo College of Medicine, Shandong University, Jinan 250031, China

## Abstract

Dysregulation of matrix metalloproteinase- (MMP-) 9 is implicated in the pathogenesis of acute lung injury (ALI). However, it remains controversial whether MMP-9 improves or deteriorates acute lung injury of different etiologies. The receptor for advanced glycation end products (RAGE) plays a critical role in the pathogenesis of acute lung injury. MMPs are known to mediate RAGE shedding and release of soluble RAGE (sRAGE), which can act as a decoy receptor by competitively inhibiting the binding of RAGE ligands to RAGE. Therefore, this study is aimed at clarifying whether and how pulmonary knockdown of MMP-9 affected sepsis-induced acute lung injury as well as the release of sRAGE in a murine cecal ligation and puncture (CLP) model. The analysis of GEO mouse sepsis datasets GSE15379, GSE52474, and GSE60088 revealed that the mRNA expression of MMP-9 was significantly upregulated in septic mouse lung tissues. Elevation of pulmonary MMP-9 mRNA and protein expressions was confirmed in CLP-induced mouse sepsis model. Intratracheal injection of MMP-9 siRNA resulted in an approximately 60% decrease in pulmonary MMP-9 expression. It was found that pulmonary knockdown of MMP-9 significantly increased mortality of sepsis and exacerbated sepsis-associated acute lung injury. Pulmonary MMP-9 knockdown also decreased sRAGE release and enhanced sepsis-induced activation of the RAGE/nuclear factor-*κ*B (NF-*κ*B) signaling pathway, meanwhile aggravating sepsis-induced oxidative stress and inflammation in lung tissues. In addition, administration of recombinant sRAGE protein suppressed the activation of the RAGE/NF-*κ*B signaling pathway and ameliorated pulmonary oxidative stress, inflammation, and lung injury in CLP-induced septic mice. In conclusion, our data indicate that MMP-9-mediated RAGE shedding limits the severity of sepsis-associated pulmonary edema, inflammation, oxidative stress, and lung injury by suppressing the RAGE/NF-*κ*B signaling pathway via the decoy receptor activities of sRAGE. MMP-9-mediated sRAGE production may serve as a self-limiting mechanism to control and resolve excessive inflammation and oxidative stress in the lung during sepsis.

## 1. Introduction

Sepsis, which is defined as a syndrome of systemic inflammation in response to infection, often leads to life-threatening multiorgan dysfunction [1]. In particular, the lungs are susceptible to sepsis and more than 50% septic patients develop acute lung injury (ALI) or acute respiratory distress syndrome (ARDS) [2, 3]. However, despite the development of clinical practices in critical care medicine for the treatment of ALI/ARDS, there still remains a mortality rate as high as 45%, and the prognosis of ALI/ARDS in patients with sepsis is still poor [4, 5]. It is requisite to understand the precise mechanisms underlying sepsis-induced lung injuries and discover effective new therapeutic methods to improve survival in ARDS patients, especially those with sepsis.

Matrix metalloproteinases (MMPs) are a family of zinc-dependent endoproteases that are involved in degradation and remodeling of the extracellular matrix (ECM) [6]. Among MMPs, MMP-9 is of particular interest, because it is elevated in patients with ALI and ARDS and correlates positively with lung injury severity [7–10]. However, it remains controversial whether MMP-9 improves or deteriorates acute lung injury of different etiologies. For example, inhibition of MMP-9 attenuates neutrophilic inflammation and pulmonary injury in ventilator-induced lung injury model [11] and improves survival in rodent models of cecal ligation and puncture- (CLP-) induced sepsis [12]. In contrast, MMP-9 deficiency is found to worsen the inflammatory responses induced by abdominal sepsis, mechanical ventilation, and allergen challenge, suggesting that MMP-9 protects against inflammatory lung injury [13–15]. Hence, further investigations are warranted to explore whether and how MMP-9 is involved in the pathophysiology of sepsis-induced ALI/ARDS.

The receptor for advanced glycation end products (RAGE) is a cell-surface membrane protein of the immunoglobulin superfamily expressed by many cell types including endothelial cells, macrophages/monocytes, and epithelium [16, 17]. RAGE plays a critical role in inflammation and oxidative stress processes and has been implicated in the pathogenesis of various lung diseases including pulmonary fibrosis, asthma, pneumonia, and acute lung injury [18]. For example, RAGE/nuclear factor-*κ*B (NF-*κ*B) signaling mediates lipopolysaccharide- (LPS-) induced ALI in neonate rat model [19]. Anti-RAGE antibodies protect mice from lethality in a CLP model of polymicrobial sepsis [20].

Soluble receptor for advanced glycation end product (sRAGE), the isoform of RAGE found in human serum, is formed by proteolytic cleavage of RAGE on cell surfaces [21, 22]. Because this isoform lacks a transmembrane domain, sRAGE is secreted and acts as a decoy receptor [23]. A previous study has shown that MMPs mediate RAGE shedding and release of sRAGE from lung epithelial cells after LPS challenge [24]. In addition, sRAGE has been proved to significantly attenuate lung inflammation in LPS-induced lung injury model [25]. Notably, MMP-9 has been demonstrated to be involved in PMA and TNF-*α*-induced RAGE shedding [23, 26]. These findings raise an intriguing possibility that MMP-9 may exert protective effects against sepsis-induced ALI via promoting the release of sRAGE.

Therefore, this study first clarified whether and how pulmonary knockdown of MMP-9 affected sepsis-induced acute lung injury as well as the release of sRAGE in a murine CLP model. It has been well recognized that RAGE mediates inflammation and oxidative stress mainly by activating the NF-*κ*B-dependent signaling pathways. We then examined the effects of MMP-9 knockdown and sRAGE on sepsis-induced pulmonary inflammation, oxidative stress, and the related signaling pathways.

## 2. Materials and Methods

### 2.1. Microarray Data Collection

The Gene Expression Omnibus (GEO) databases GSE15379, GSE52474, and GSE60088 (http://www.ncbi.nlm.nih.gov/geo/) were used to analyze the expression of MMP-9 in sepsis-induced lung injury. The search terms used were sepsis and lung.

### 2.2. Animals

Male Institute of Cancer Research (ICR) mice weighing approximately 25-30 g were used in this study. Animals were housed in cages and given access to food and water ad libitum at a constant ambient temperature and humidity with 12 h day-night cycling. All experimental procedures were approved by the Animal Ethics Committee of Xinhua Hospital, Shanghai Jiaotong University School of Medicine.

### 2.3. Cecal Ligation and Puncture-Induced Sepsis Model and sRAGE Treatment

The CLP model is widely used and known to closely mimic septic human patients. We performed the CLP model primarily based on procedures of Rittirsch et al. [27]. Briefly, mice were anesthetized by intraperitoneal administration of mixed ketamine (70 mg/kg) and xylazine (10 mg/kg). Then, a 1 cm midline incision was made in the lower abdomen. Cecum was identified and ligated at half distance between the distal pole and base of the cecum; cecal puncture involves a through and through puncture (two holes) with a 21-gauge needle. Be careful when extruding a droplet of feces through each puncture site to ensure patency. Then, the cecum was returned to the abdominal cavity; the peritoneum and skins were closed by simple sutures. Animals were rewarmed until fully conscious and then returned to free food and water. The sham group received exactly the same procedures except for the CLP. Anesthesia and analgesics were used for all surgical experiments to avoid unnecessary suffering of the mice. The response of mice during experiments was monitored carefully to maintain adequate depth of anesthesia. Mice were humanely sacrificed by CO_2_ inhalation when they met the following humane-endpoint criteria: prostration, significant body weight loss, breathing difficulty, rotational motion, and body temperature drop [28]. Recombinant sRAGE protein (Adio Bo Biological Ltd., Beijing, China) was administrated intratracheally 10 minutes before the initiation of CLP, and lung tissues were collected 24 h after CLP.

### 2.4. Histopathological Evaluation of the Lung Tissue

The lungs were fixed with 4% formalin, embedded in paraffin, and cut into 4 *μ*m slices. After hematoxylin and eosin (H&E) staining, pathological changes of lung tissues were observed under a light microscope. Lung injury score was assessed by two blinded pathologists who are expert in lung pathology. To examine the extent of lung injury, we considered its five pathological features: (1) presence of exudates, (2) infiltration of neutrophils, (3) intra-alveolar hemorrhage/debris, (4) cellular infiltration, and (5) cellular hyperplasia. The severity of each of these pathological features was evaluated by a score indicating 0 as absent/none, 1 as slight, 2 as to show mild, 3 for moderate, 4 as moderate to severe, and finally 5 for severe injury [29, 30].

### 2.5. Immunofluorescence

Paraffin sections (4 *μ*m) of lung tissues were rehydrated and microwaved in citric acid buffer to retrieve antigens. After incubation with 10% BSA for 1 h, the sections were incubated with primary antibodies against F4/80 (cat. no. GB11027, 1 : 300; Servicebio Technology, Wuhan, China) and 8-hydroxy-2′-deoxyguanosine (8-OHdG) (cat. no. ab48508, 1 : 200; Abcam, Cambridge, MA, USA) overnight. Subsequently, tissue slices were incubated with secondary antibody corresponding to each primary antibodies (CY3: goat anti-rabbit, cat. no. GB21303, 1 : 300; Servicebio; Alexa Fluor 488: goat anti-mouse, cat. no. GB25301, 1 : 400; Servicebio) in dark for 1 h and counterstained with 4′,6-diamidino-2-phenylindole (DAPI) (cat. no. C1005, Beyotime Institute of Biotechnology, Shanghai, China). Stained slices were observed using an Olympus fluorescence microscope.

### 2.6. Quantitative Real-Time Polymerase Chain Reaction (PCR) Analysis

Total RNA was extracted from lung tissues using RNAiso Plus reagent (Takara Biotechnology, Dalian, China) and processed into cDNA according to the manufacturer's protocol. Quantitative real-time PCR was performed by monitoring the increase in fluorescence using 2× One Step SYBR® Green Mix^a^ (Vazyme Biotech, Nanjing, Jiangsu, China) with the use of StepOnePlus system (Applied Biosystems, Foster City, CA). The comparative threshold cycle (Ct) method with arithmetic formulae (2^-*ΔΔ*Ct^) was used to determine the relative quantization of gene expression. The primer sequences of related genes are shown in Supplemental Table 1.

### 2.7. Western Blotting

Proteins were extracted from lung tissues (25 mg) using RIPA (Beyotime) containing protease and phosphatase inhibitor cocktail (Beyotime) according to the manufacturer's instructions. Protein concentrations were determined by the BCA protein assay kit (Beyotime). Equal amounts of proteins (40 *μ*g) were separated with 10% SDS-PAGE, transferred to PVDF membrane (Millipore, Billerica, MA, USA), and blocked with 5% nonfat milk. Membranes were incubated overnight with specific primary antibody against MMP-9 (Abcam), RAGE (Abcam), NF-*κ*B p65 (Cell Signaling Technology, Danvers, MA, USA), phosphorylated p65 (Cell Signaling Technology), inhibitor of *κ*B-*α* (I*κ*B-*α*) (Cell Signaling Technology), and *β*-actin (Sigma-Aldrich, Mo, USA).

### 2.8. In Vivo MMP-9 siRNA Transfection

The small interfering RNA (siRNA) sequences directed against mouse MMP-9 were constructed by GenePharma Corp (Shanghai, China). To locally knockdown pulmonary MMP-9 expression, 1 mg/kg MMP-9 siRNA or control siRNA was diluted in vivo jetPEI® (Polyplus, NY, USA). Aliquots of 30 *μ*l siRNA/jetPEI mixture were injected intratracheally into the lung 48 hours before the onset of sepsis. The siRNA sequences used in this study were provided in Supplemental Table 2.

### 2.9. Bronchoalveolar Lavage

Bronchoalveolar lavage (BAL) fluid was collected via tracheal catheter using three sequential 1 ml aliquots of sterile saline. Cell pellets were collected by centrifugation (4°C, 1500 rpm, 10 min) and then resuspended in PBS for total cell counting. Protein concentration was determined by BCA protein assay kit.

### 2.10. ELISA

The concentrations of IL-6 (Cusabio Biotech, Wuhan, China), MCP-1 (Cusabio Biotech), MDA (Elabscience Biotechnology, Wuhan, China), and sRAGE (R&D Systems, Minneapolis, MN, USA) in the lung tissues and BAL fluid were measured by ELISA kits according to the instruction recommended by the manufacturers.

### 2.11. Statistical Analysis

The results are expressed as mean ± SEM. Statistical significance in experiments comparing only two groups was determined by two-tailed Student's *t*-test. The comparison among multiple groups was estimated by one-way analysis of variance followed by post hoc analysis using Student-Newman-Keuls test. To determine statistical significance between survival curves, the Kaplan–Meier test was used. A *p* value of <0.05 was considered significant. All statistical analyses were done with SPSS 22.0 (SPSS Inc., Chicago, USA).

## 3. Results

### 3.1. Pulmonary Level of MMP-9 Is Upregulated in the CLP-Induced Sepsis Model

**Figure 1:**
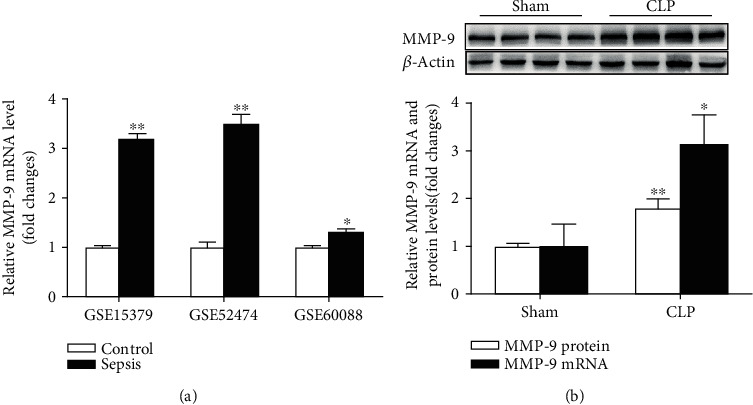
Pulmonary level of MMP-9 is upregulated in the CLP-induced sepsis model. (a) mRNA expression levels of MMP-9 in septic mouse lung tissues. Data were obtained from the following Gene Expression Omnibus datasets: GSE15379, GSE52474, and GSE60088. (b) Mice were subjected to CLP or sham surgery. mRNA and protein expression levels of MMP-9 were verified by RT-PCR and western blotting in lung tissues (*n* = 7). Representative protein bands were presented on the top of the histograms. Data are presented as the mean ± SEM. ^∗^*p* < 0.05 and ^∗∗^*p* < 0.01 vs. the control or sham group. MMP-9: matrix metalloproteinase-9; CLP: cecal ligation and puncture.

To evaluate the MMP-9 expression in the lung of septic mice, we analyzed the gene expression profiles of mouse sepsis-induced lung injury datasets GSE15379 [31], GSE52474 [32], and GSE60088 [33]. Information regarding the mouse sepsis datasets is presented in Supplementary Table 3. Collectively, the analysis of these data revealed that MMP-9 mRNA expression was significantly upregulated in lung tissues of septic mice (Figure 1(a)). To verify these results, pulmonary MMP-9 expression was determined by RT-PCR and western blotting in the CLP-induced mouse sepsis model. As shown in Figure 1(b), both mRNA and protein levels of MMP-9 were significantly increased in the CLP group compared with those in the sham group.

### 3.2. Pulmonary Knockdown of MMP-9 Increases Mortality of Sepsis and Exacerbates Sepsis-Associated Acute Lung Injury

**Figure 2:**
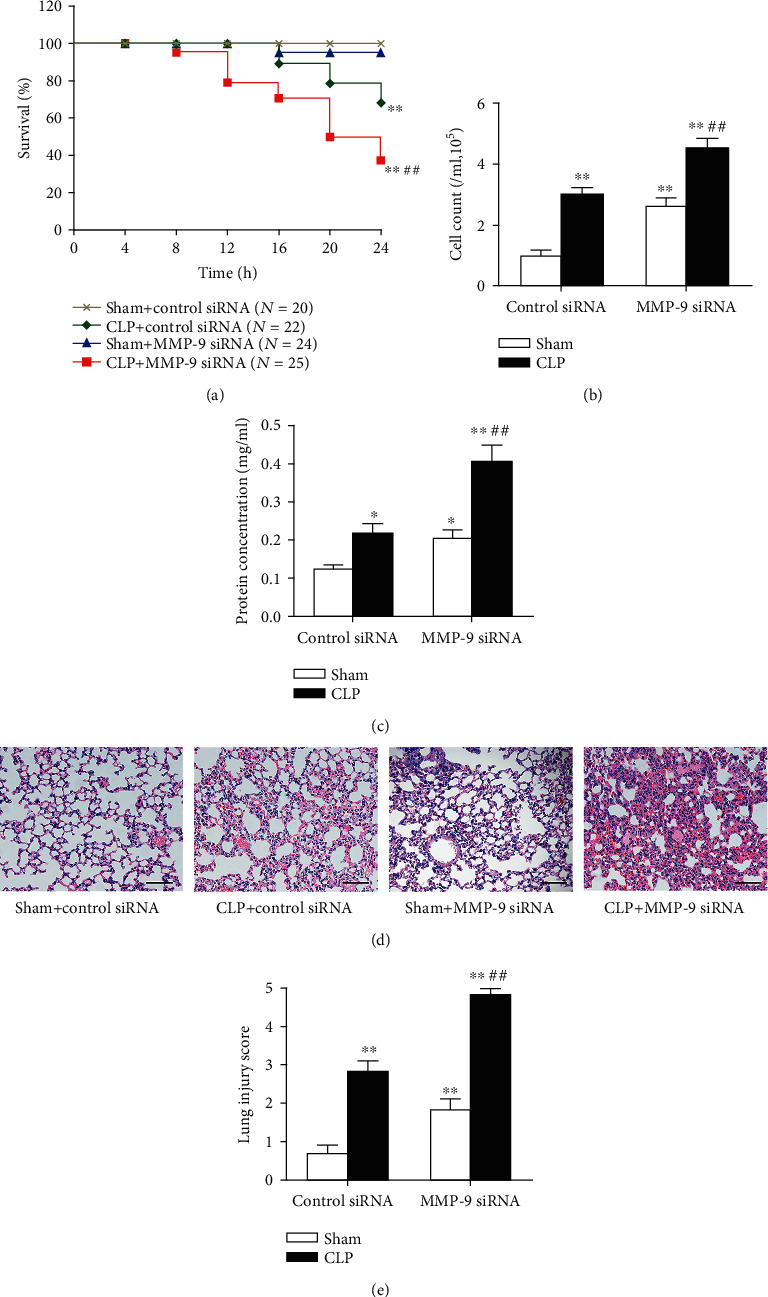
Pulmonary knockdown of MMP-9 increases mortality of sepsis and exacerbates sepsis-associated acute lung injury. Mice were intratracheally injected with MMP-9 siRNA or control siRNA (1 mg/kg). Forty-eight hours later, mice were subjected to CLP or sham surgery. (a) Mice were carefully monitored for 24 h after CLP or sham surgery, and survival rates at the indicated times were recorded. (b) Cell count and (c) protein concentration in BAL fluid. Histopathological examination using hematoxylin and eosin staining (d). Original magnification, ×200. Scale bars correspond to 50 *μ*m. The severity of lung injury was scored by a pathologist blinded to group allocation (e). Data are presented as the mean ± SEM (*n* = 7). ^∗^*p* < 0.05 and ^∗∗^*p* < 0.01 vs. the sham+control siRNA group. ^##^*p* < 0.01 vs. the CLP+control siRNA group. BAL: bronchoalveolar lavage.

We then investigated whether MMP-9 upregulation was involved in sepsis-induced lung injury. As shown in Supplementary Figure 1, intratracheal injection of MMP-9 siRNA (1 mg/kg) resulted in an approximately 60% decrease in pulmonary MMP-9 expression. We found that survival of MMP-9 siRNA-treated mice that underwent CLP operation was significantly lower than survival of control siRNA-treated mice that underwent CLP operation (Figure 2(a)). The lung injury was evaluated at 24 h after challenge with CLP or a sham operation. As shown in Figures 2(b) and 2(c), mice subjected to CLP operation exhibited significant lung injury as indicated by increases in cell count and protein content in BAL fluid. MMP-9 siRNA-treated mice had a significantly higher BAL cell count and protein levels than control siRNA-treated mice after CLP challenge. Histological characteristics showed that CLP challenge led to obvious interstitial tissue edema and inflammatory cell infiltration both in control siRNA-treated and MMP-9 siRNA-treated mice. Pulmonary knockdown of MMP-9 led to more severe lung damage than control siRNA-treated mice after CLP challenge, which was also demonstrated by quantitative analysis of lung injury score (Figures 2(d) and 2(e)). Notably, we found that pulmonary knockdown of MMP-9 *per se* led to a mild but significant lung injury as illustrated by elevated BAL cell count and protein levels, as well as increased lung injury score.

### 3.3. Pulmonary Knockdown of MMP-9 Decreases sRAGE Release and Enhances Sepsis-Induced Activation of the RAGE/NF-*κ*B Signaling Pathway in Lung Tissues

**Figure 3:**
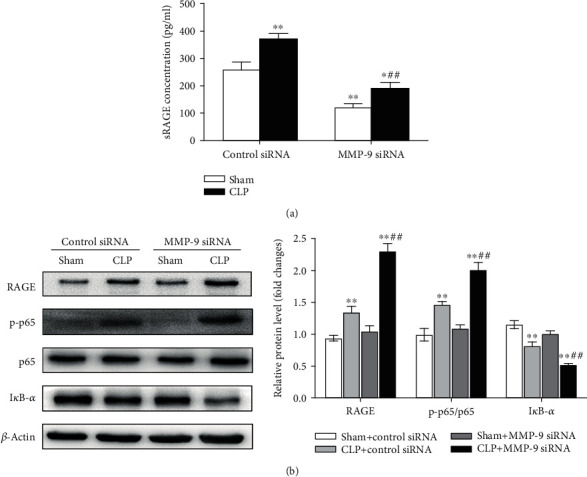
Pulmonary knockdown of MMP-9 decreases sRAGE release and enhances sepsis-induced activation of the RAGE/NF-*κ*B signaling pathway in lung tissues. Mice were intratracheally injected with MMP-9 siRNA or control siRNA (1 mg/kg). Forty-eight hours later, mice were subjected to CLP or sham surgery. (a) sRAGE concentrations in BAL fluid. (b) Protein levels of RAGE, phosphorylated NF-*κ*B p65 subunit (p-p65), and I*κ*B-*α* were determined by western blot analysis. p-p65 levels were normalized to total p65 expression. Representative protein bands were presented on the left of the histograms. Data are presented as the mean ± SEM (*n* = 7). ^∗^*p* < 0.05 and ^∗∗^*p* < 0.01 vs. the sham+control siRNA group. ^##^*p* < 0.01 vs. the CLP+control siRNA group. BAL: bronchoalveolar lavage.

Previous studies have shown that sheddase MMP-9 can cleave transmembrane RAGE to produce a soluble form of RAGE [23, 26]. As shown in Figure 3(a), we found that sRAGE release in BAL fluid was obviously enhanced in the CLP group compared with that in the sham group. Pulmonary knockdown of MMP-9 significantly decreased sRAGE release in the sham group. Moreover, CLP-induced increase of sRAGE release in BAL fluid was largely prevented by MMP-9 knockdown.

Accumulating studies have shown the activation of RAGE and the downstream NF-*κ*B signaling pathway during the development of sepsis-induced acute lung injury [34–36]. We found that protein levels of RAGE and phosphorylated NF-*κ*B p65 subunit in lung tissues were increased, whereas I*κ*B-*α* was decreased in the CLP group compared with the sham group. Notably, pulmonary knockdown of MMP-9 significantly aggravated the CLP-induced RAGE/NF-*κ*B activation as indicated by augmented RAGE and phosphorylated p65 levels and decreased I*κ*B-*α* expression in lung tissues of septic mice (Figures 3(b) and 3(c)).

### 3.4. Pulmonary Knockdown of MMP-9 Aggravates Sepsis-Induced Oxidative Stress and Inflammation in Lung Tissues

**Figure 4:**
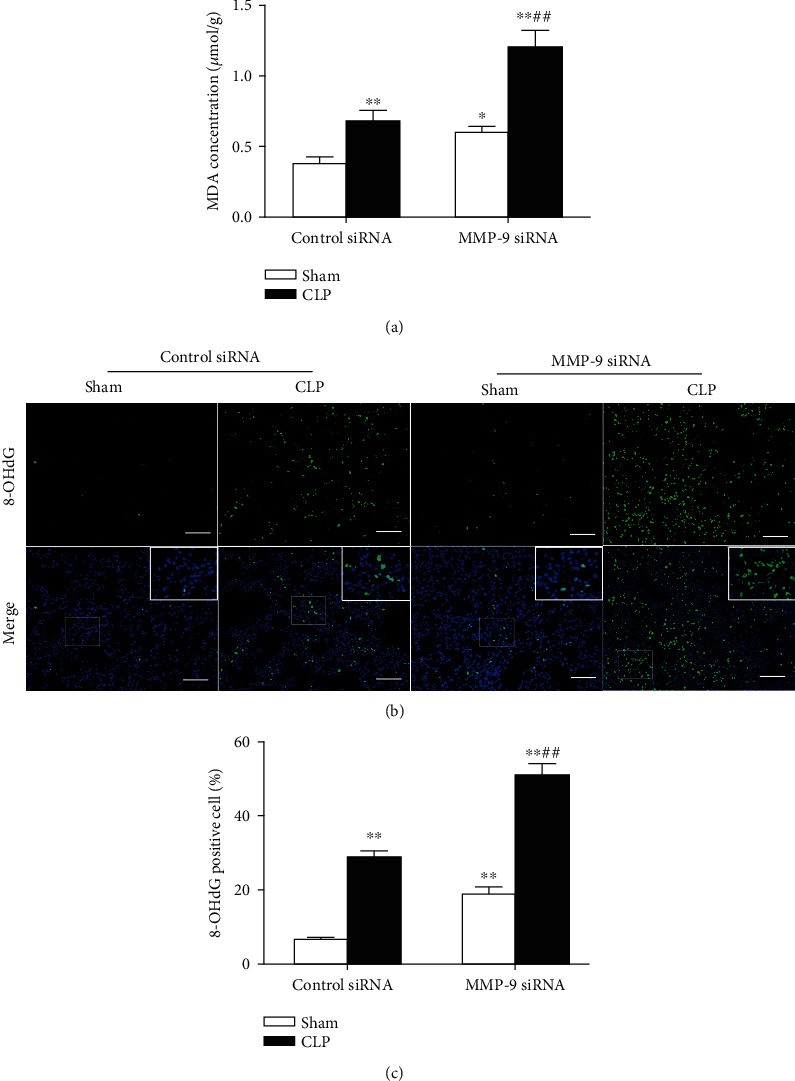
Pulmonary knockdown of MMP-9 aggravates sepsis-induced oxidative stress in lung tissues. Mice were intratracheally injected with MMP-9 siRNA or control siRNA (1 mg/kg). Forty-eight hours later, mice were subjected to CLP or sham surgery. (a) MDA levels in lung tissues. (b, c) 8-OHdG immunoreactivity (green) was measured as the index of oxidative DNA damage. Nuclei were counterstained with 4′,6-diamidino-2-phenylindole (DAPI) (blue). Areas in white boxes were shown enlarged (b). Original magnification, ×200. Scale bars correspond to 50 *μ*m. (c) The ratio of 8-OHdG-positive cells to the total cell number (%). Data are presented as the mean ± SEM (*n* = 7). ^∗^*p* < 0.05 and ^∗∗^*p* < 0.01 vs. the sham+control siRNA group. ^##^*p* < 0.01 vs. the CLP+control siRNA group. 8-OHdG: 8-hydroxy-2′-deoxyguanosine.

Activation of the NF-*κ*B signaling pathway plays a critical role in RAGE-mediated oxidative stress and inflammation [37, 38]. We then investigated the effect of pulmonary knockdown of MMP-9 on sepsis-induced oxidative stress and inflammation. MDA content and 8-OHdG immunoreactivity were measured as the index of membrane lipid peroxidation activity and oxidative DNA damage, respectively. As shown in Figure 4, significant increases in lung MDA level and 8-OHdG-positive cells were observed in the CLP group compared with the sham group. Pulmonary knockdown of MMP-9 significantly aggravated the CLP-induced pulmonary oxidative stress as indicated by augmented MDA levels and 8-OHdG immunoreactivity in lung tissues of septic mice. In addition, it was found that pulmonary knockdown of MMP-9 *per se* led to mild but significant increases in lung MDA level and 8-OHdG immunoreactivity.

**Figure 5:**
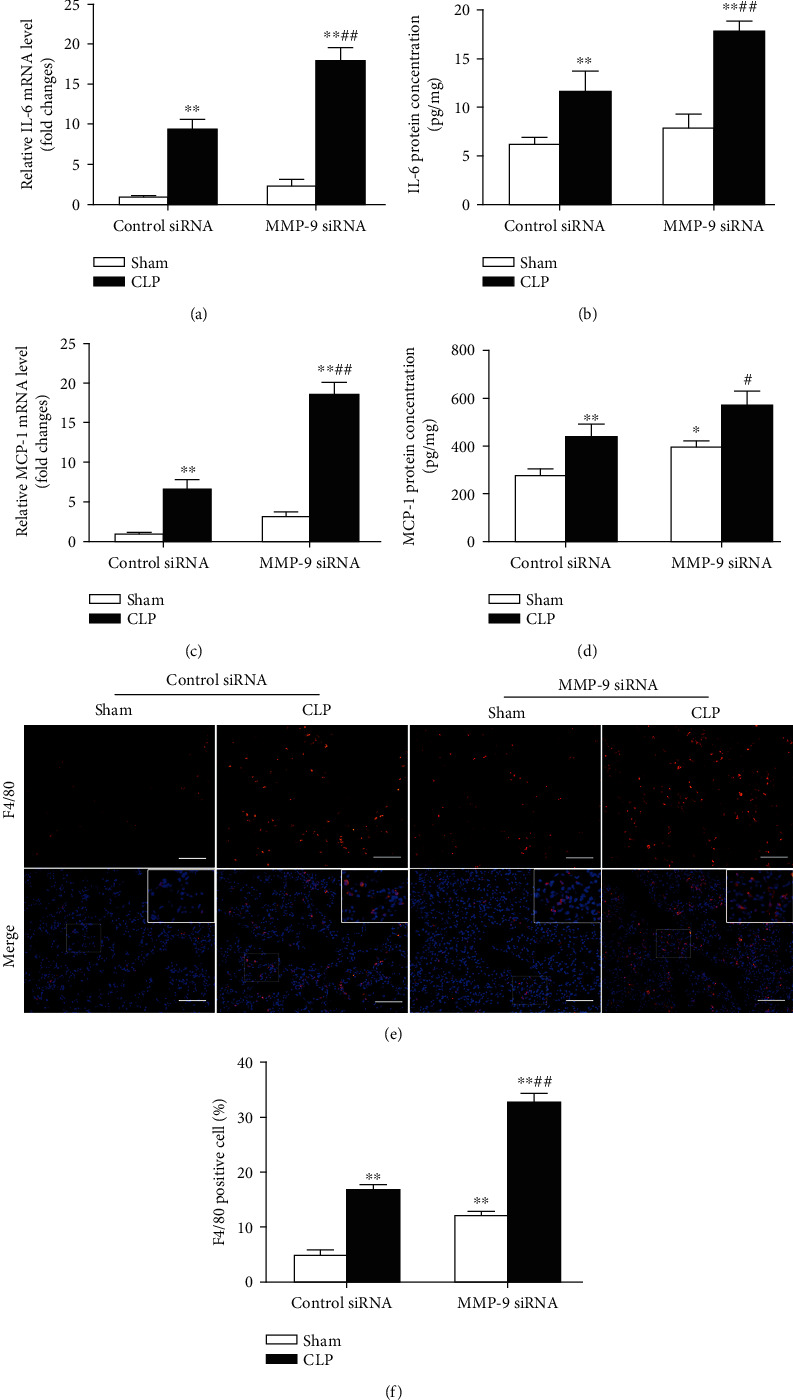
Pulmonary knockdown of MMP-9 aggravates sepsis-induced inflammation in lung tissues. Mice were intratracheally injected with MMP-9 siRNA or control siRNA (1 mg/kg). Forty-eight hours later, mice were subjected to CLP or sham surgery. Levels of (a) IL-6 mRNA, (b) IL-6 protein, (c) MCP-1 mRNA, and (d) MCP-1 protein in lung tissues. (e, f) Lung tissues were stained with fluorophore-labeled antibodies against macrophage marker F4/80 (red). Nuclei were counterstained with 4′,6-diamidino-2-phenylindole (DAPI) (blue). Original magnification, ×200. Scale bars correspond to 50 *μ*m (e). (f) The proportion of F4/80-positive cells in total cells (%). Data are presented as the mean ± SEM (*n* = 7). ^∗^*p* < 0.05 and ^∗∗^*p* < 0.01 vs. the sham+control siRNA group. ^#^*p* < 0.05 and ^##^*p* < 0.01 vs. the CLP+control siRNA group.

We then investigated the effect of pulmonary knockdown of MMP-9 on the proinflammatory cytokine IL-6, chemokine MCP-1, and macrophage infiltration in lung tissues. As shown in Figure 5, CLP operation caused significant increases in mRNA and protein levels of IL-6 (Figure 5(a)) and MCP-1 (Figure 5(b)), which were significantly aggravated by MMP-9 knockdown. There were very few F4/80-positive staining cells in the lung tissues of the sham group. Mice that underwent CLP operation developed severe infiltration of F4/80+ macrophages. The quantification analysis showed that the percentage of F4/80+ macrophages in lung tissues was further augmented by MMP-9 knockdown (Figure 5(f)). Moreover, pulmonary knockdown of MMP-9 *per se* led to mild but significant increases in lung MCP-1 protein level and macrophage infiltration.

Taken together, these results indicate that pulmonary knockdown of MMP-9 aggravates sepsis-induced oxidative stress and inflammation in lung tissues.

### 3.5. Administration of sRAGE Attenuates Oxidative Stress, Inflammatory Response, and Lung Injury Induced by Intrapulmonary Knockdown of MMP-9

**Figure 6:**
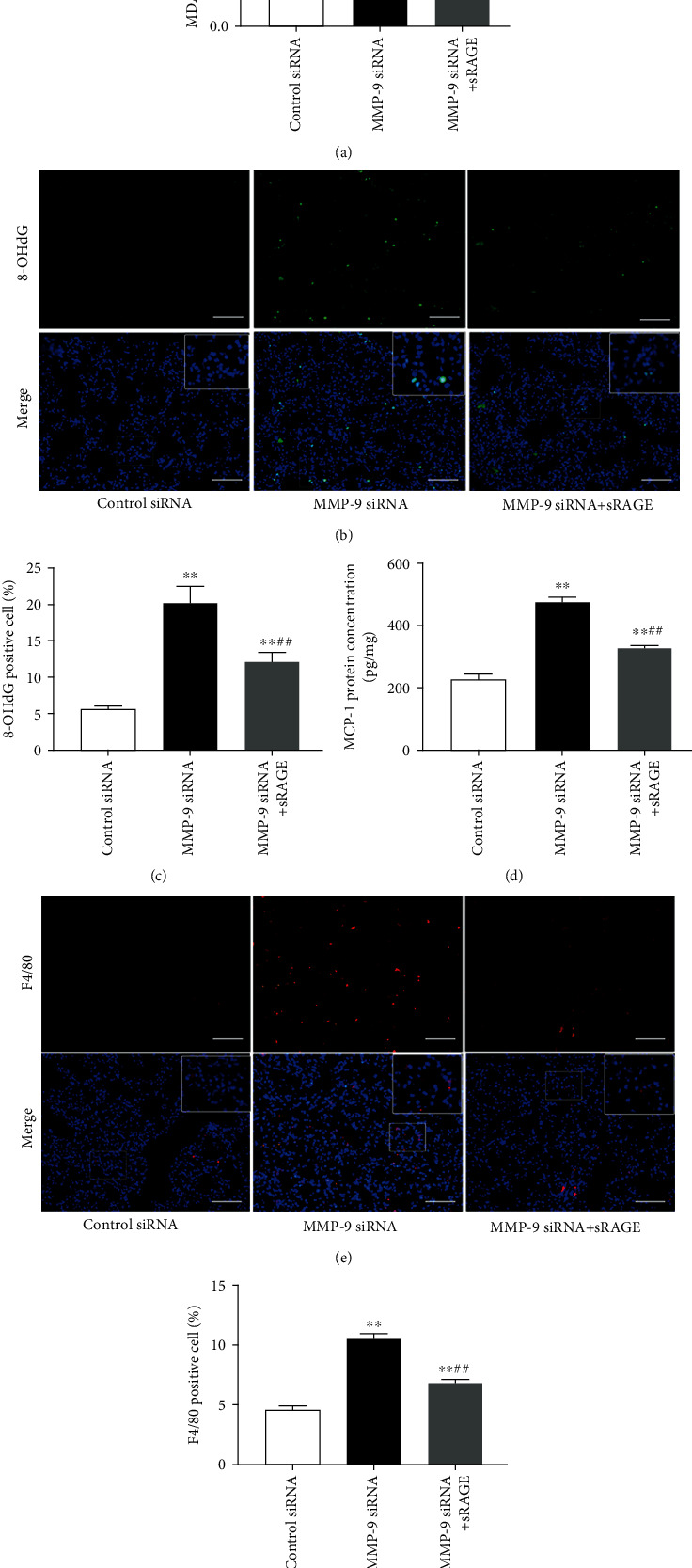
Administration of sRAGE attenuated oxidative stress and inflammatory response induced by intrapulmonary knockdown of MMP-9. Mice were intratracheally injected with MMP-9 siRNA or control siRNA (1 mg/kg). Forty-eight hours later, mice were administrated with recombinant sRAGE protein. (a) MDA levels in lung tissues. (b) 8-OHdG immunoreactivity (green) was measured as the index of oxidative DNA damage. (c) The ratio of 8-OHdG-positive cells to the total cell number (%). (d) MCP-1 levels in lung tissues. (e) Lung tissues were stained with fluorophore-labeled antibodies against macrophage marker F4/80 (red). (f) The proportion of F4/80-positive cells in total cells (%). Nuclei were counterstained with 4′,6-diamidino-2-phenylindole (DAPI) (blue). Areas in white boxes were shown enlarged. Original magnification, ×200. Scale bars correspond to 50 *μ*m. Data are presented as the mean ± SEM (*n* = 7). ^∗∗^*p* < 0.01 vs. the control siRNA group. ^##^*p* < 0.01 vs. the MMP-9 siRNA group.

**Figure 7:**
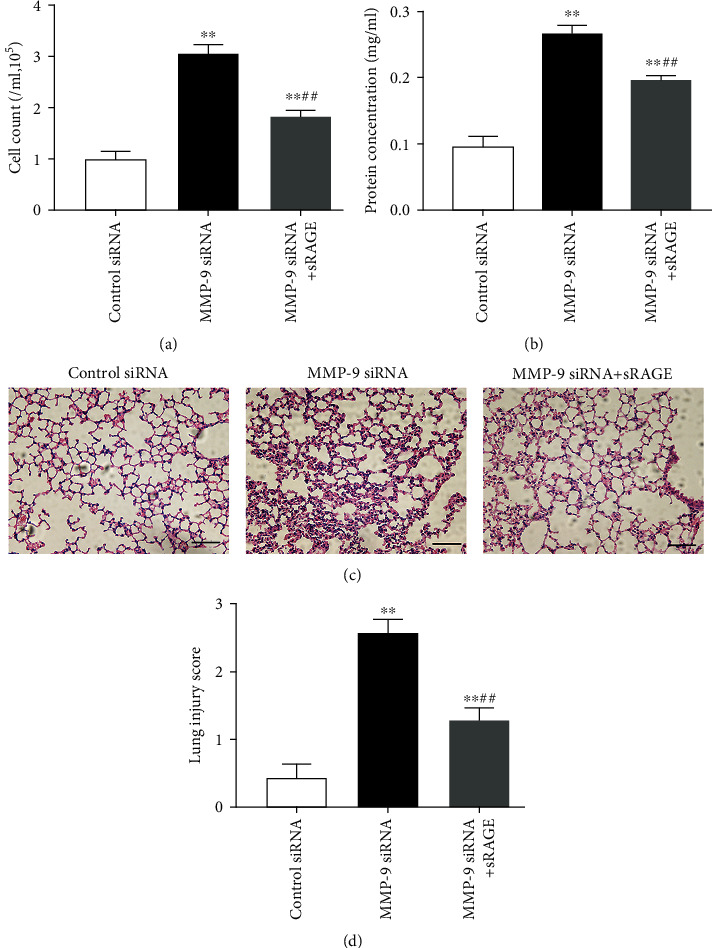
Administration of sRAGE attenuated lung injury induced by intrapulmonary knockdown of MMP-9. Mice were intratracheally injected with MMP-9 siRNA or control siRNA (1 mg/kg). Forty-eight hours later, mice were administrated with recombinant sRAGE protein. (a) Cell count and (b) protein concentration in BAL fluid. (c) Representative hematoxylin- and eosin-stained sections of lung tissue were shown. (d) The severity of lung injury from different groups was scored by a pathologist blinded to group allocation. Original magnification, ×200. Scale bar indicates 50 *μ*m. Data are presented as the mean ± SEM (*n* = 7). ^∗∗^*p* < 0.01 vs. the control siRNA group. ^##^*p* < 0.01 vs. the MMP-9 siRNA group.

There is growing evidence that sRAGE acts as a decoy receptor and exerts protective effects against acute lung injuries [25, 39]. In the present study, we found that administration of recombinant sRAGE protein significantly attenuated oxidative stress and inflammatory responses induced by intrapulmonary knockdown of MMP-9, as evidenced by the reduction in MDA level (Figure 6(a)), 8-OHdG-positive cells (Figures 6(b) and 6(c)), MCP-1 level (Figure 6(d)), and macrophage infiltration (Figures 6(e) and 6(f)) in lung tissues. Furthermore, intrapulmonary knockdown of MMP-9-induced increases in BAL cell count (Figure 7(a)) and protein levels (Figure 7(b)) as well as lung injury score (Figures 7(c) and 7(d)) was also markedly alleviated by administration of sRAGE.

### 3.6. Administration of sRAGE Suppresses the Activation of the RAGE/NF-*κ*B Signaling Pathway and Ameliorates Oxidative Stress and Inflammation in Lung Tissues of CLP-Induced Septic Mice

**Figure 8:**
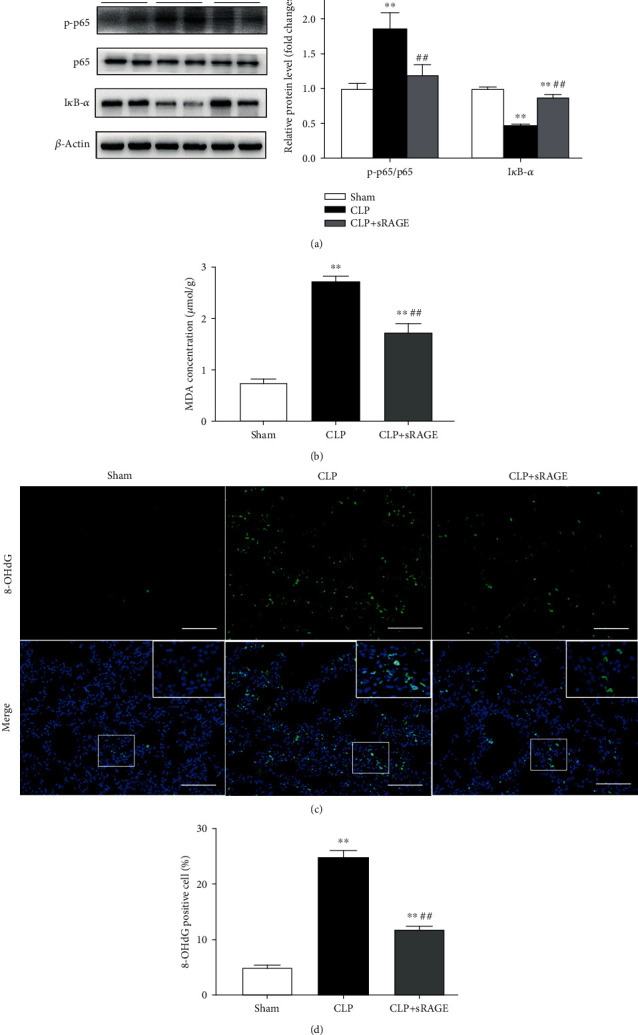
Administration of sRAGE suppresses the activation of the RAGE/NF-*κ*B signaling pathway and ameliorates oxidative stress in lung tissues of CLP-induced septic mice. Mice were subjected to CLP or sham surgery. Recombinant sRAGE protein (200 *μ*g/kg) was intratracheally injected immediately before the onset of sepsis, with an equivalent volume of saline intratracheally injected in other groups. (a) Protein levels of phosphorylated NF-*κ*B p65 subunit (p-p65) and I*κ*B-*α* were determined by western blot analysis. p-p65 levels were normalized to total p65 expression. Representative protein bands were presented on the left of the histograms. (b) MDA levels in lung tissues. (c, d) 8-OHdG immunoreactivity (green) was measured as the index of oxidative DNA damage. Nuclei were counterstained with 4′,6-diamidino-2-phenylindole (DAPI) (blue). Areas in white boxes were shown enlarged (c). Original magnification, ×200. Scale bars correspond to 50 *μ*m. (d) The ratio of 8-OHdG-positive cells to the total cell number (%). Data are presented as the mean ± SEM (*n* = 7). ^∗∗^*p* < 0.01 vs. the sham group; ^##^*p* < 0.01 vs. the CLP group.

It has been well recognized that sRAGE can act as a decoy receptor by competitively inhibiting the binding of RAGE ligands to RAGE, accordingly attenuating the downstream signaling pathways [25, 40]. As shown in Figure 8(a), the present study found that the CLP-induced increase of phosphorylated NF-*κ*B p65 subunit was largely reduced, whereas the inhibition of I*κ*B-*α* was profoundly prevented by intratracheal instillation of recombinant sRAGE protein (200 *μ*g/kg). These findings indicate that administration of sRAGE can suppress the activation of the RAGE/NF-*κ*B signaling pathway in lung tissues of CLP-induced septic mice.

**Figure 9:**
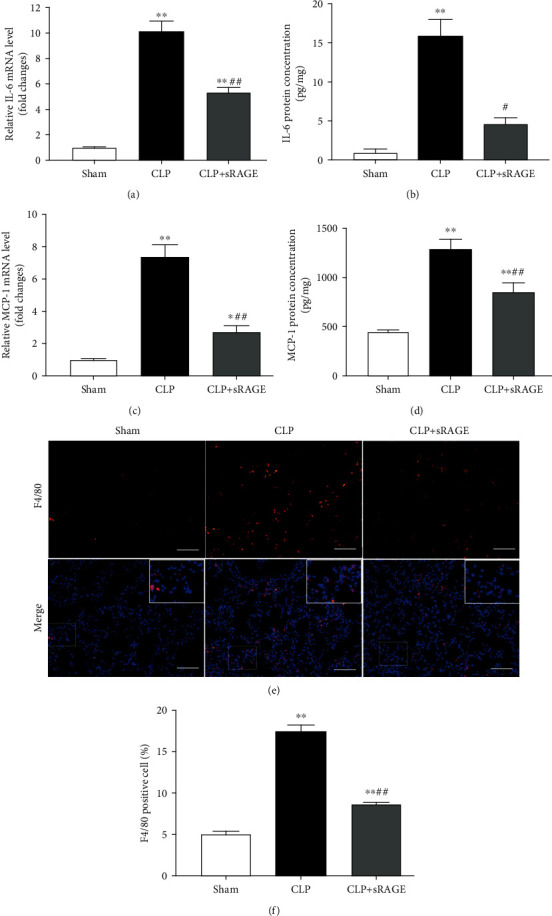
Administration of sRAGE suppresses inflammation in lung tissues of CLP-induced septic mice. Mice were subjected to CLP or sham surgery. Recombinant sRAGE protein (200 *μ*g/kg) was intratracheally injected immediately before the onset of sepsis, with an equivalent volume of saline intratracheally injected in other groups. Levels of (a) IL-6 mRNA, (b) IL-6 protein, (c) MCP-1 mRNA, and (d) MCP-1 protein in lung tissues. (e, f) Lung tissues were stained with fluorophore-labeled antibodies against macrophage marker F4/80 (red). Nuclei were counterstained with 4′,6-diamidino-2-phenylindole (DAPI) (blue). Original magnification, ×200. Scale bars correspond to 50 *μ*m (e). (f) The proportion of F4/80-positive cells in total cells (%). Data are presented as the mean ± SEM (*n* = 7). ^∗^*p* < 0.05 and ^∗∗^*p* < 0.01 vs. the sham group; ^#^*p* < 0.05 and ^##^*p* < 0.01 vs. the CLP group.

Intratracheal instillation of recombinant sRAGE protein significantly ameliorated oxidative stress in lung tissues of CLP-induced septic mice, as indicated by reduced lung MDA level and 8-OHdG-positive cells (Figures 8(b)–8(d)). Moreover, CLP-induced increases in pulmonary levels of IL-6 and MCP-1 as well as macrophage infiltration were also significantly attenuated by intratracheal instillation of sRAGE (Figure 9).

### 3.7. Administration of sRAGE Attenuates Sepsis-Associated Acute Lung Injury

**Figure 10:**
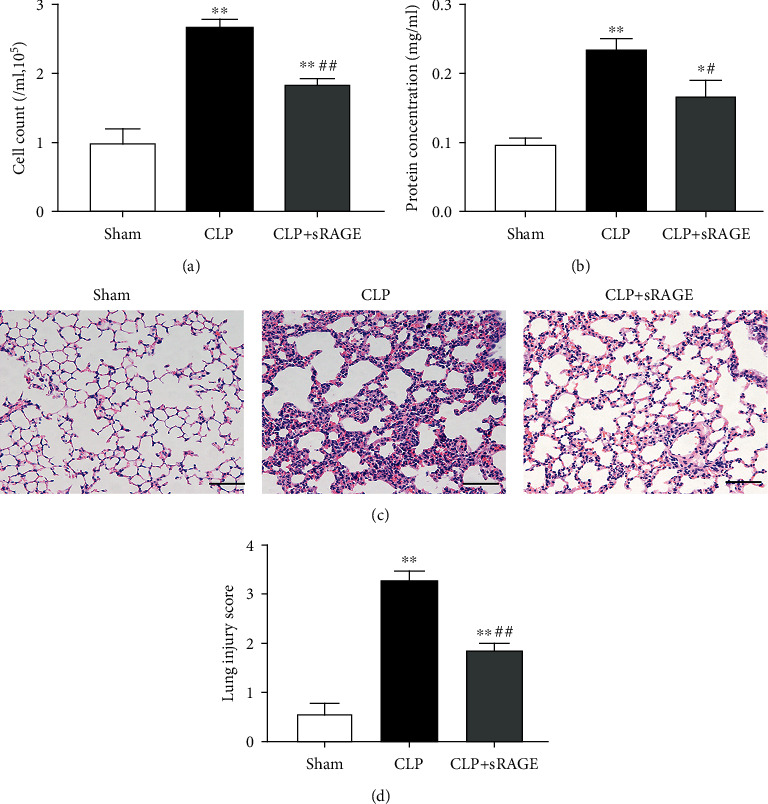
Administration of sRAGE attenuates sepsis-associated acute lung injury. Mice were subjected to CLP or sham surgery. Recombinant sRAGE protein (200 *μ*g/kg) was intratracheally injected immediately before the onset of sepsis, with an equivalent volume of saline intratracheally injected in other groups. (a) Cell count and (b) protein concentration in BAL fluid. Histopathological examination using hematoxylin and eosin staining (c). Original magnification, ×200. Scale bars correspond to 50 *μ*m. The severity of lung injury was scored by a pathologist blinded to group allocation (d). Data are presented as the mean ± SEM (*n* = 7). ^∗^*p* < 0.05 and ^∗∗^*p* < 0.01 vs. the sham group; ^#^*p* < 0.05 and ^##^*p* < 0.01 vs. the CLP group.

As shown in Figures 10(a) and 10(b), we found that intratracheal instillation of recombinant sRAGE protein significantly reduced cell count and protein content levels in BAL fluid of CLP-induced septic mice. Histological analysis of lung sections revealed that CLP-induced lung injury was significantly attenuated by intratracheal instillation of sRAGE, as indicated by improved interstitial edema, enlarged alveolar air space, and decreased infiltration of inflammatory cells. As shown in Figure 10(c), the lung injury score was reduced from 3.29 ± 0.53 (CLP only) to 1.86 ± 0.38 (CLP plus sRAGE). However, administration of recombinant sRAGE resulted in a slight but not significant improvement in survival of CLP-induced septic mice (Supplementary Figure 2), although it attenuated sepsis-associated acute lung injury.

## 4. Discussion

Dysregulation of MMP-9 has been reported during the pathogenesis of acute lung injury [41]. In the present study, the gene expression profiles from three datasets generated in lung tissues obtained from septic mice were analyzed and exhibited elevated mRNA expression levels of MMP-9. Using a CLP-induced sepsis model, it was observed that both mRNA and protein expression levels of MMP-9 in lung tissues were increased in the CLP group compared to the sham group. Consistent with our results, it has been demonstrated that pulmonary MMP-9 is upregulated in experimental ALI animal models induced by cardiopulmonary bypass (CPB) [42] and in patients with sepsis [9, 43].

There are conflicting data regarding the roles of MMP-9 in the development and full manifestation of acute lung injury of diverse etiologies. For example, MMP-9 knockout mice develop more severe distant organ damage during infection [13] and enhanced allergen-induced airway inflammation [15]. In contrast, nonspecific inhibition of MMPs prevents neutrophilic inflammation in ventilator-induced lung injury model [43] and improves survival in sepsis-associated lung injury model [12]. Moreover, Rahman et al. report that pulmonary neutrophil infiltration, edema formation, and lung injury are markedly decreased in septic mice lacking MMP-9 [44]. Considering that MMP-9 is a widely expressed matrix metalloproteinase, global blockade or knockout of MMP-9 may not well reflect the local effects of MMP-9 in the development of acute lung injuries. Thus, siRNA-based technology was used in the present study to locally knockdown pulmonary MMP-9 expression. Our results demonstrated that intrapulmonary knockdown of MMP-9 significantly aggravated sepsis-associated ALI as indicated by increased pulmonary edema, inflammation, oxidative stress, and lung injury score. Notably, local suppression of MMP-9 in the lung led to a significant increase in sepsis-induced mortality. Taken together, these findings suggest that pulmonary upregulation of MMP-9 may be recognized as part of a self-protective response to sepsis-associated ALI.

MMP-9 belongs to a family of zinc-dependent endopeptidases that can cleave a variety of substrates, ranging from extracellular matrix to cell surface proteins and a number of cytokines [6, 45]. MMP-9 has been found to cleave IL-1*β*, thus negatively regulating the activity of IL-1*β* [46]. In abdominal sepsis, MMP-9 controls the shedding of platelet-derived CD40L, which is known to regulate neutrophil recruitment and lung damage in sepsis [44]. Furthermore, MMP-9 is identified to be involved in RAGE shedding stimulated by PMA [26] or TNF-*α* [23], which leads to a release of sRAGE, a decoy receptor neutralizing RAGE ligands. In the present study, we found that intrapulmonary knockdown of MMP-9 significantly decreased sRAGE release and enhanced sepsis-induced activation of the RAGE signaling pathway in lung tissues. In addition, administration of sRAGE suppressed the activation of the RAGE signaling pathway and attenuated ALI induced by intrapulmonary knockdown of MMP-9 and CLP-induced sepsis. These findings suggest that MMP-9-mediated RAGE shedding may contribute to the self-protective effects of pulmonary MMP-9 upregulation against lung injuries during sepsis.

Activation of the RAGE-dependent NF-*κ*B signaling pathway has been implicated in acute lung injury of diverse etiologies including acid aspiration [47], endotoxin [48], hyperoxia [49, 50], traumatic brain injury [51], and sepsis [34, 35]. The NF-*κ*B signaling has been well recognized as a critical inducer of inflammatory responses [52]. Evidence from both *in vitro* and *in vivo* studies has shown that downregulation of RAGE is associated with decreased levels of proinflammatory cytokines, concomitant with reduced NF-*κ*B signaling in alveolar type I epithelial cells [53] and lung tissues [54]. In recent years, accumulating studies report that activation of RAGE also induces oxidative stress via NF-*κ*B-dependent pathways [38, 55]. In line with these findings, this study found that sepsis-associated inflammation and oxidative stress in the lung were accompanied by activation of the RAGE/NF-*κ*B signaling pathway. In addition, intrapulmonary knockdown of MMP-9 aggravated, whereas administration of recombinant sRAGE attenuated sepsis-associated activation of the RAGE/NF-*κ*B signaling pathway, consequently resulting in the exacerbation or improvement of pulmonary inflammation and oxidative stress, respectively. Our results suggest that MMP-9-mediated RAGE shedding might limit the severity of sepsis-associated lung damage by suppressing the RAGE/NF-*κ*B signaling pathway in the lung via the decoy receptor activities of sRAGE.

sRAGE has been reported to exert potent protection against lung injuries induced by LPS [56], acid instillation [39, 54], sepsis [57], and mechanical ventilation [58]. However, the mechanisms responsible for the pulmonary protective effects of sRAGE remain largely unknown. sRAGE has been shown to attenuate LPS-induced inflammation, hyperpermeability, and apoptosis in the lung by inhibiting NF-*κ*B activation [25]. In acid-injured lungs, administration of sRAGE exerts protective effects through a decrease in alveolar type 1 epithelial cell injury as shown by restored alveolar fluid clearance (AFC) and lung aquaporin- (AQP-) 5 expression [54]. In this study, we provided the *in vivo* evidence that sRAGE treatment alleviated CLP-induced RAGE expression, NF-*κ*B p65 subunit phosphorylation, downregulation of I*κ*B*α*, macrophage infiltration, production of proinflammatory cytokines, and oxidative stress in lung tissues. Collectively, these findings suggest that inhibition of the RAGE/NF-*κ*B signaling pathway as well as pulmonary inflammation and oxidative stress may contribute to the protection against CLP-induced lung injury afforded by sRAGE.

Notably, administration of recombinant sRAGE had no significant effect on the mortality of CLP-induced septic mice, although it suppressed sepsis-induced activation of RAGE/NF-*κ*B, inflammation, and oxidative stress in lung tissues. There is growing evidence that administration of recombinant sRAGE can prevent a variety of organ injuries including myocardial ischemia/reperfusion injury [59, 60], chronic intermittent hypoxia-induced renal injury [61], CCl_4_-induced liver injury [62], and acute lung injuries [39, 54]. On the other hand, recent clinical trials report that increased levels of sRAGE are associated with higher risks of mortality in frail older adults [63], hemodialysis and peritoneal dialysis patients [64], cardiovascular diseases [65, 66], and ARDS [67]. Collectively, these findings let us suggest that increased MMP-9-mediated sRAGE production may represent a self-protective mechanism in response to sepsis, whereas exogenous enhancement of sRAGE levels is not sufficient to improve survival.

## 5. Conclusions

In conclusion, this study demonstrated that pulmonary upregulation of MMP-9 might be recognized as part of a self-protective response to sepsis-associated lung injury. MMP-9-mediated RAGE shedding limited the severity of sepsis-associated pulmonary edema, inflammation, oxidative stress, and lung injury by suppressing the RAGE/NF-*κ*B signaling pathway via the decoy receptor activities of sRAGE. Our data indicate that MMP-9-mediated sRAGE production may serve as a self-limiting mechanism to control and resolve excessive inflammation and oxidative stress in the lung during sepsis.

## Data Availability

All raw data will be available upon request to the corresponding author from this manuscript.

## Supplementary Materials

Supplementary Materials Supplementary Materials Table S1: primer sequences used in the real-time quantitative PCR. Table S2: small interfering RNA sequences used in the present study. Table S3: mouse sepsis datasets: timepoints of analysis, number of mice, and type of samples in each group. Figure S1: pulmonary knockdown of MMP-9. Mice were intratracheally injected with MMP-9 siRNA or control siRNA (1 mg/kg). Forty-eight hours later, the protein expression level of MMP-9 was determined by western blot analysis. Relative densitometry of the MMP-9 protein band over *β*-actin was shown in the bar graph. The representative protein bands were presented on the top of the bar graphs. Data are expressed as means ± SEM (*n* = 7). ^∗∗^*p* < 0.01 vs. the control siRNA group. Figure S2: sRAGE did not significantly improve the survival rate of CLP-induced septic mice. Recombinant sRAGE protein (200 *μ*g/kg) was intratracheally injected into the lung of mice, and then, mice were subjected to CLP or sham surgery. Survival rates at the indicated times were recorded.
