# Supplementary material for: Upregulation of Matrix Metalloproteinase-9 Protects against Sepsis-Induced Acute Lung Injury via Promoting the Release of Soluble Receptor for Advanced Glycation End Products
Source: Oxid Med Cell Longev. 2021 Feb 10;2021:8889313. doi: 10.1155/2021/8889313 (PMC7889353; doi:10.1155/2021/8889313)
Supplement: Supplementary Materials — Table S1: primer sequences used in the real-time quantitative PCR. Table S2: small interfering RNA sequences used in the present study. Table S3: mouse sepsis datasets: timepoints of analysis, number of mice, and type of samples in each group. Figure S1: pulmonary knockdown of MMP-9. Mice were intratracheally injected with MMP-9 siRNA or control siRNA (1 mg/kg). Forty-eight hours later, the protein expression level of MMP-9 was determined by western blot analysis. Relative densitometry of the MMP-9 protein band over β-actin was shown in the bar graph. The representative protein bands were presented on the top of the bar graphs. Data are expressed as means ± SEM (n = 7). ∗∗p < 0.01 vs. the control siRNA group. Figure S2: sRAGE did not significantly improve the survival rate of CLP-induced septic mice. Recombinant sRAGE protein (200 μg/kg) was intratracheally injected into the lung of mice, and then, mice were subjected to CLP or sham surgery. Survival rates at the indicated times were recorded. [file 8889313.f1.doc]

Supplemental Digital Content

**Upregulation of matrix metalloproteinase-9 protects against sepsis-induced acute lung injury via promoting the release of soluble receptor for advanced glycation end products**

Hui Zhang^1^*, Yan-Fei Mao^1^*, Ying Zhao^2^, Dun-Feng Xu^1^, Yan Wang^1^, Chu-Fan Xu^1^, Wen-Wen Dong^1^, Xiao-Yan Zhu^3^, Ding Ning^4^, Lai Jiang^1^, Yu-Jian Liu^2^

1-Department of Anesthesiology and Surgical Intensive Care Unit, Xinhua Hospital, Shanghai Jiaotong University School of Medicine, Shanghai, 200092.

2-School of Kinesiology, The Key Laboratory of Exercise and Health Sciences of Ministry of Education Shanghai University of Sport, Shanghai, 200438.

3-Department of Physiology, Navy Medical University, Shanghai, 200433.

4-Department of Anesthesiology, Shandong Provincial Third Hospital, Cheeloo College of Medicine, Shandong University, Jinan, 250031.

*These authors contributed equally to this work and should be considered as co-first authors

**Corresponding Authors:**

Prof. Yu-Jian Liu

School of Kinesiology, The Key Laboratory of Exercise and Health Sciences of Ministry of Education Shanghai University of Sport, 200 Hengren Road, Shanghai 200438, China. Email: liuyujian@sus.edu.cn;

Prof. Lai Jiang

Department of Anesthesiology and Surgical Intensive Care Unit, Xinhua Hospital, Shanghai Jiaotong University School of Medicine, 1665 Kongjiang Road ,Shanghai 200092, China. Email: jianglai@xinhuamed.com.cn

**Supplemental Table S1. Primer sequences used in the real-time quantitative PCR**

Gene Forward (5’-3’) Reverse (5’-3’)

MMP-9 TTCGCAGACCAAGAGGGTTTTC AAGATGTCGTGTGAGTTCCAGGGC

IL-6 CTGCAAGAGACTTCCATCCAG AGTGGTATAGACAGGTCTGTTGG

MCP-1 CCCACTCACCTGCTGCTACT TCTGGACCCATTCCTTCTTG

β-actin CTGTATGCCTCTGGTCGTAC TGATGTCACGCACGATTTCC

**Supplemental Table S2. Small interfering RNA sequences used in the present study**

sense antisense

MMP-9 siRNA 5’-GCCAGACACUAAAGGCCAUTT-3’ 5’-AUGGCCUUUAGUGUCUGGCTT-3’

Control siRNA 5’-UUCUCCGAACGUGUCACGUTT-3’ 5’-ACGUGACACGUUCGGAGAATT-3’

**Supplemental Table S3. Mouse sepsis datasets: timepoints of analysis, number of mice and type of samples in each group.**

| GSE series | GSE15379 | GSE52474 | GSE60088 |
| --- | --- | --- | --- |
| Timepoints | 8h | 24h | 8h |
| Number of mice (control:sepsis) | 3:3 | 4:3 | 4:5 |
| Type of samples | Lung tissues | Lung tissues | Lung tissues |

**
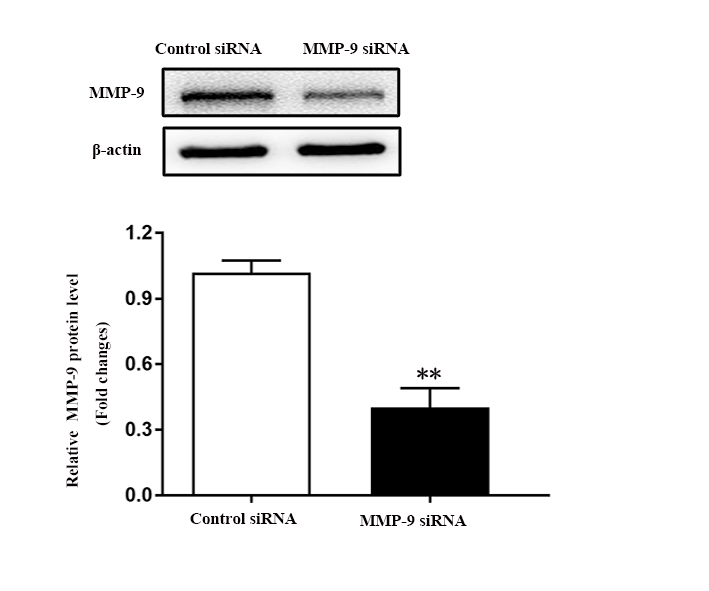
**

**Supplemental Figure S1. Pulmonary knockdown of MMP-9.** Mice were intratracheally injected with MMP-9 siRNA or control siRNA (1 mg/kg). Forty eight hours later, the protein expression level of MMP-9 was determined by western blot analysis. Relative densitometry of the MMP-9 protein band over β-actin was shown in bar graph. The representative protein bands were presented on the top of the bar graphs. Data are expressed as means ± SEM (n=7).** p< 0.01 vs. control siRNA group.


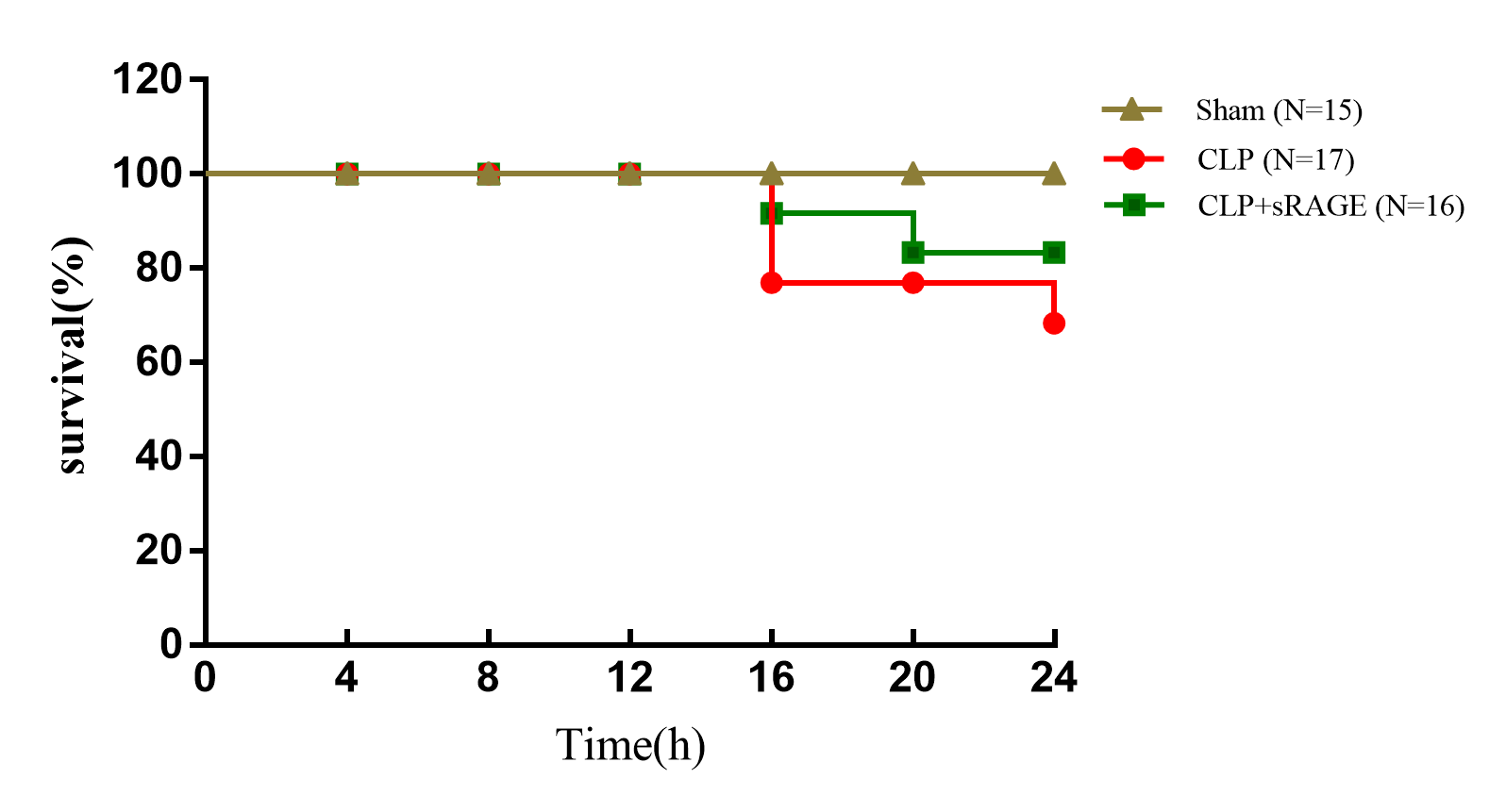


**Supplemental Figure S2. sRAGE did not significantly improve survival rate of CLP-induced septic mice.** Recombinant sRAGE protein (200 μg/kg) was intratracheally injected into the lung of mice and then mice were subjected to CLP or Sham surgery. Survival rates at the indicated times were recorded.
